# Effect of moderate hyperventilation and induced hypertension on cerebral tissue oxygen saturation in comatose post-cardiac arrest patients treated with hypothermia

**DOI:** 10.1186/cc12249

**Published:** 2013-03-19

**Authors:** T Suys, N Sala, M Oddo

**Affiliations:** 1 Lausanne University Hospital, Lausanne, Switzerland

## Introduction

Maintenance of adequate brain perfusion is an essential component of post-resuscitation care. Mean arterial pressure (MAP) and PaCO_2 _are important determinants of brain perfusion; however, no precise guidelines exist for optimal MAP and PaCO_2 _targets in comatose post-cardiac arrest (CA) patients.

## Methods

Using NIRS, we examined changes in non-invasive cerebral tissue oxygen saturation (SctO_2_) following moderate hyperventilation (HV) and induced hypertension (IH) in comatose CA patients treated with therapeutic hypothermia (TH). A prospective pilot study including comatose patients successfully resuscitated from out-of-hospital CA treated with TH (33°C for 24 hours, using cold saline and surface cooling device), monitored for continuous SctO_2 _with the Foresight NIRS system (CAS Medical Systems, Branford, CT, USA). Moderate hyperventilation was induced for approximately 30 minutes by decreasing PaCO_2 _from ~40 to ~30 mmHg, at stable MAP. After PaCO_2 _normalization, MAP was increased from ~70 to ~90 mmHg by intravenous infusion of norepinephrine, at stable PaCO_2_. Effects of MV and IH on SctO_2 _were analyzed with a paired *t *test.

## Results

**Figure 1:**
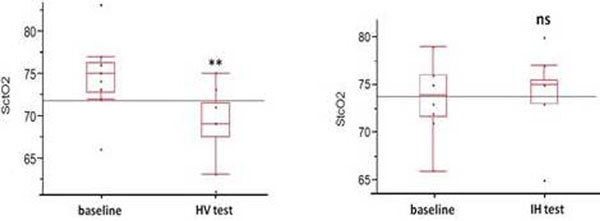
**Changes in SctO_2 _after moderate HV and IH tests (*n = *10 patients)**.

Ten patients (mean age, 69.5; mean time to ROSC, 19 minutes) were studied during the stable TH maintenance phase. Results are summarized in Figure 1. MV was associated with a significant reduction of SctO_2 _from baseline (75% (73 to 76) to 69% (67.5 to 71.5), *P <*0.001).

No significant changes in SctO_2 _were found after IH (74 (72 to 76) vs. 75 (73 to 75.5), *P *= 0.24).

## Conclusion

Moderate HV was associated with significant reduction in cerebral saturation, whilst IH may be detrimental after CA and TH, whilst increasing MAP to supranormal levels with vasopressors does not improve cerebral oxygenation. These data stress the importance of strict control of PaCO_2 _following CA and TH to avoid secondary cerebral ischemic insults.

